# Effectiveness of the Brown Lacewing, *Sympherobius barberi* Banks as a Biological Control Agent of the Asian Citrus Psyllid *Diaphorina citri* Kuwayama

**DOI:** 10.3389/fpls.2020.567212

**Published:** 2020-10-14

**Authors:** Azhar A. Khan, Muhammad Afzal, Phil Stansly, Jawwad A. Qureshi

**Affiliations:** ^1^Southwest Florida Research and Education Center, Entomology and Nematology Department, University of Florida, Gainesville, FL, United States; ^2^College of Agriculture, Bahauddin Zakariya University, Multan, Pakistan; ^3^Department of Entomology, University of Sargodha, Sargodha, Pakistan

**Keywords:** Asian citrus psyllid, citrus greening, biological control, predator, lacewings

## Abstract

The Asian citrus psyllid (ACP) *Diaphorina citri* Kuwayama is an economically important pest of citrus because it vectors the causal pathogens of huanglongbing (HLB) or citrus greening disease. Biological control is an important component of citrus pest management but requires consistent strengthening of its impact on pest complex. The brown lacewing *Sympherobius barberi* Banks is a known predator of several insect pests from Asia, Europe, and America. However, there is not much information about its effectiveness against *D. citri*. We evaluated *S. barberi* against the *D. citri* and frozen eggs of the Mediterranean flour moth *Ephestia kuehniella*, the latter is a common diet used for rearing predators in laboratories. Adult *S. barberi* successfully fed on *D. citri* eggs and nymphs under both light and dark conditions. *Diaphorina citri* was also suitable for the development and reproduction of *S. barberi* except for slightly prolonged larval development compared with *E. kuehniella* diet. The egg hatch from the total number of eggs laid on *D. citri* and *E. kuehniella* diets averaged 65% and 52%, respectively. Females laid 64% eggs on dimpled white paper compared to 36% combined on plain paper and leaves of citrus, orange jasmine, eggplant and cantaloupe. *Sympherobius barberi* released at densities of 2–6 adults against eggs and nymphs of *D. citri* on infested orange jasmine plants in the cages provided a reduction of 43–81% in the number of provided eggs or nymphs. In the field tests on *D. citri* infested citrus trees, reduction averaged 35% in five cohorts in which developing colonies of 28–32 nymphs were provided to one *S. barberi* per cage. Findings suggest the significant potential of *S. barberi* as a predator of *D. citri* and to contribute to reducing huanglongbing.

## Introduction

The Asian citrus psyllid (ACP) *Diaphorina citri* Kuwayama (Hemiptera: Liviidae) is a primary vector of the causal pathogens of huanglongbing (HLB) also known as citrus greening disease. It is an economically important pest of citrus in HLB affected regions (Catling, 1970). First described in Taiwan (Kuwayama, 1908), *D. citri* was later found in Punjab Pakistan and the rest of the region (Hussain and Nath, 1927) and reported from Brazil in 1940 (Lima, 1947) and Florida in 1998 (Halbert, 1998). Huanglongbing in Florida was detected in 2005 (Halbert, 2005) 1 year after its identification from Brazil in 2004 (Texeira et al., 2005).

Several members of the order Neuroptera are known bio-control agents of multiple pests and are distributed all over the world. Chrysopidae and Hemerobiidae families received significant attention as bio-control agents because several species are voracious feeders of soft-bodied insects (Balduf, 1939; New, 1975).

Hemerobiidae family, with approximately 27 genera and 560 described species, includes many brown lacewings, which are predators of a wide range of insect pests (Oswald, 1993, 1994; New, 2001; Grimaldi and Engel, 2005). Most species of hemerobiids are predaceous in both imaginal and preimaginal stages (Miller and Cave, 1987). Adults of several species are generalist predators, and many are habitat-specific (Killington, 1937; Monserrat and Marín, 1996). Brown lacewings are known as natural enemies of the several insect pests in classical biological control (Sato and Takada, 2004). Both larvae and adults of several species are efficient predators on eggs and immature stages of several pests. Due to their prolonged longevity, high consumption rates, and high reproduction rate, they are established as important bio-control agents in the agro-ecosystem (MacLeod and Stange, 2005). *Sympherobius barberi* is native to Continental US, Canada and Mexico, while geographically it is distributed throughout the North America, Europe, and Northern Asia (excluding China), Middle America, Oceania and South America (Penny, 1977; Penny et al., 1997). Pacheco-Rueda et al. (2011) evaluated *S. barberi* against *Dactylopius opuntiae*. They reported a net reproductive rate (R_o_) of 36.6, a daily intrinsic rate of increase (r_m_) of 0.081, a generation time (T) of 44.27 days, and a finite reproductive rate (λ) of 1.084 for *S. barberi*.

Most soft-bodied insects and their eggs are preferred food for brown lacewing. Cutright (1923) observed the larva of the brown lacewing; *Micromus posticus* Walker consumes 41 aphids throughout its life. Yayla and Satar (2012) found out that larvae of *Sympherobius pygmaeus* Rambur on the diet of *Planococcus citri* developed in 31 days at 25°C and female laid maximum of 258 eggs.

Our objectives for these studies were (1) to investigate the development, reproduction and predation potential of *S. barberi* on the diet of *D. citri*, in comparison with frozen eggs of the Mediterranean flour moth *Ephestia kuehniella* (Lepidoptera: Pyralidae), and (2) to evaluate *S. barberi* for reducing *D. citri* populations under greenhouse and field conditions. Eggs of *E. kuehniella* is a conventional diet for rearing several predatory species. The findings of this study may improve our understanding of utilizing *S. barberi* for augmentative biological control purposes in citrus crops worldwide, particularly in systems where increased chemical control is negatively impacting naturally occurring predators.

## Materials and Methods

### Study Location, Insects, and Experimental Conditions

Colonies of *D. citri* and *S. barberi* were maintained and experiments conducted at the Southwest Florida Research and Education Center (SWFREC) of the University of Florida-IFAS, Gainesville, FL, United States. *Diaphorina citri* colony was maintained on orange jasmine *Murraya paniculata* (L.), in a climate-controlled glasshouse set at 28°C. *Murraya paniculata* is a close relative of citrus, and one of the preferred hosts of *D. citri*. *Sympherobius barberi* adults originally obtained from Foothill Agricultural Research, Inc. (550 W Foothill Pkwy, Corona, CA, United States) were used to maintain its colony on frozen eggs of *E. kuehniella* (Koppert Biological Systems, Romulus, MI, United States) and nectar honey. The colony used several groups of 20 adults per 3-L ventilated plastic jar. The frozen eggs of *E. kuehniella* and nectar honey were provided for food and a small cube of moist sponge for water. Shoots of *M. paniculata* and pieces of paper towel were provided as a substrate for oviposition. Eggs laid on paper towel or leaves were then placed in 20 ml (5-DRAM) snap cap vials and observed for hatching. The colony was maintained in an incubator (Percival, Model I36LLC8, Percival Scientific, Inc., Perry, IA, United States) set to a photoperiod of 16:8 (L:D) at 25°C. Upon hatching, neonates were collected daily, and experiments conducted in the incubator and conditions described for the colony.

### Laboratory Experiments

#### Feeding

Twenty-four hours after emergence, adult *S. barberi* were used to test for feeding on *D. citri* nymphs under light and dark conditions in the growth chamber. Sixty adults of *S. barberi* were distributed randomly across three different diets (1) psyllid eggs, (2) psyllid nymphs 2^*nd*^ and 3^*rd*^ (small) instars, and (3) psyllid nymphs 4^*th*^ and 5^*th*^ (large) instars. One adult was provided with 25–30 psyllid eggs and 20 small or large nymphs on young orange jasmine shoots in the petri dish (9-cm diameter and 1.5-cm height). The petri dishes were covered with perforated parafilm to prevent the escape of nymphs and provide ventilation. The number of eggs and nymphs consumed by *S. barberi* were recorded at 6, 12, and 24 h.

#### Reproduction

**FIGURE 1:**
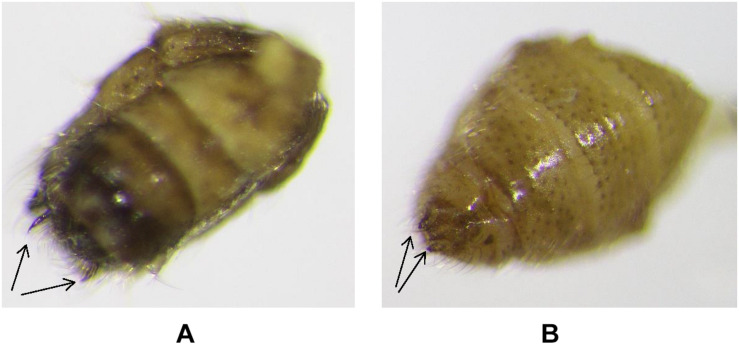
Abdominal terminallia of *S. barberi*, Male: **(A)** and Female: **(B)**. Male and female pairs for reproduction tests were separated by distinguishing genital appendages morphologically, using 14X triplet hasting magnification hand lens. Male **(A)** has flattened shaped terminalia with two claws, while Female **(B)** has tipped end with two knobs. Slide photos were taken by using Olympus SZX16 stereo microscope fixed with DP21 digital camera.

Male and female were distinguished by examining their genital appendages using the 14X Triplet Hasting Magnification hand lens. The male has flattened shaped terminalia with two claws, while the female has tipped end with two knobs (Figures 1A,B). Each pair of male and female was held in 100 ml perforated snap-cap plastic vial arena and provided with diets under three treatments to evaluate reproduction; (1) nectar honey on a paper strip (2) frozen eggs of *E. kuehniella* and (3) eggs and younger nymphs of *D. citri* on shoots of *M. paniculata*. Shoots of *M. paniculata* and dimpled white paper towel (1“X4”) were provided in the vials as a substrate for oviposition. Eggs laid by *S. barberi* were collected for 3 weeks, and food replaced every 24 h.

**FIGURE 2:**
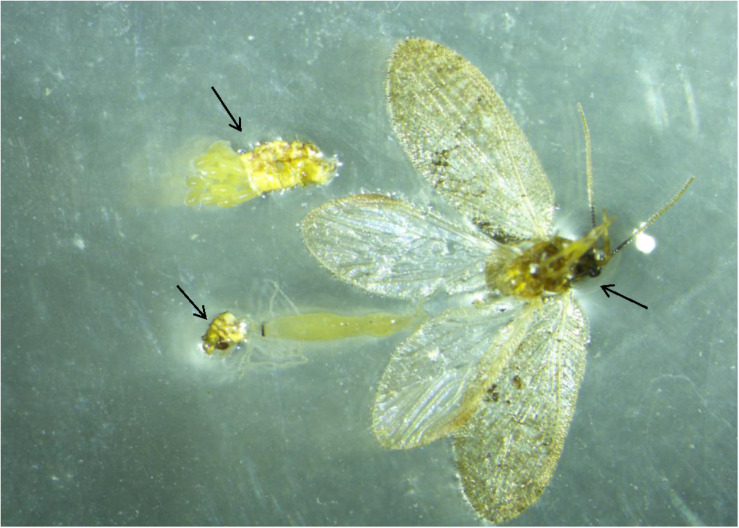
Dissected *S. barberi* female with egg cluster. Female was dissected under microscope (using fine needle/forceps) for the presence of egg cluster before and after the experiment. Female and cluster are visible in the figure. This dissection was also done to confirm the morphological differences in the male and female genital appendages. Slide photo was taken by using an Olympus SZX16 stereo microscope fixed with digital camera DP21.

Eggs from each male-female pair were kept in 20 ml (5-DRAM) snap cap vials in the growth chamber under conditions described above and examined daily for hatching. Upon death, females were dissected and examined under a stereomicroscope (Olympus SZX16, SDF-PLAPO, Japan) fixed with a digital camera (DP21) to check for the presence of eggs (Figure 2). A separate experiment was conducted to test female preference to lay eggs on six different substrates including, dimpled white paper, black paper, and leaves of four different plant species, including Citrus, Orange jasmine, Eggplant, and Cantaloupe using 10 female replicates.

#### Development

Eggs obtained from the parents maintained on the diets of nectar honey, frozen eggs of *E. kuehniella*, and eggs and nymphs of *D. citri* were held separately for each diet at one egg per 20 ml (5-DRAM) snap cap vial. There were 10 replicates for each of the three diets. Upon hatching, larvae were reared on the same three diets on which their parents were maintained. Food was replaced daily and data recorded on larval survival, pupation, and adult emergence. The longevity of adult *S. barberi* was evaluated in two experiments (1) including diets of eggs of *E. kuehniella*, eggs of *D. citri*, and nymphs of *D. citri*, and (2) mixed diets including eggs plus nymphs of *D. citri* as a separate treatment, with honey and honey by itself. Twenty replicates were performed for each diet.

### Greenhouse Experiments

The potted plants of *M. paniculata* were pruned and placed individually in bugdorm cages to induce new shoots needed for psyllid reproduction and development. The bugdorm cage (58 cm × 58 cm × 66 cm) made from clear plastic included two side panels of fine Polyester netting for ventilation. Psyllid adults used in the experiment were obtained from a colony maintained on *M. paniculata*, in a glasshouse at 25 ± 2°C provided with 1000W metal-halide light set to 16: 8 (L:D). The adult psyllids of mixed gender (50 males, 50 females) were released in each experimental cage.

The plants were checked daily for oviposition by psyllids. Sixteen plants adjusted to an average of 100 eggs on 3–4 shoots/plant were moved to new cages at one per cage. The plants were assigned randomly to three release densities of *S. barberi* adults (2, 4, and 6 adults/cage) and control without any adult for a total of four replicates per predator density treatment. Eggs were counted on the plants 3 days after adult release and before hatch to assess the number consumed by the predator. The plants were kept in the same cages to evaluate *S. barberi* predation on nymphs that emerged from the viable eggs. Nymphs consumed, dead, or emerged to adults were counted at an interval of 1 day for 1 week.

### Field Experiment

Four years old 5 ft high ‘Hamlin’ citrus plants at Southwest Florida Research and Education Center, Immokalee, FL, United States were pruned to encourage the development of young shoots needed for psyllid reproduction and development. Once infested with *D. citri*, 20 shoots were assigned randomly to two groups designated for release of *S. barberi* and control with no release. The number of psyllid nymphs (mostly younger instars 2^*nd*^ and 3^*rd*^) were counted on each shoot using the14X Triplet Hasting Magnification hand lens. Shoots were enclosed with sleeve cages made from fine mesh organdy. Those assigned for the predator’s release received one adult *S. barberi* per shoot with a colony averaging 28–32 nymphs. Data regarding the consumption of psyllid nymphs and the emergence of adult psyllids in all cages were recorded at an interval of 1 day. *S. barberi* were moved to new shoots within the same age range of *D. citri* nymphs every 2–3 days until death. The previously used shoots were brought to the laboratory, and *D. citri* nymphs and adults were counted. In total, 42 nymphal colonies were used in the five cohorts, the first three cohorts with 10 nymphal colonies in each and 4^*th*^ and 5^*th*^ cohort with 7 and 5 colonies, respectively. All nymphs in the control colonies (*n* = 10) were allowed to develop to adulthood and counted in the laboratory.

### Statistical Analysis

A generalized linear model (GLM) procedure was used for Analysis of Variance (ANOVA) followed by Fisher’s least significant difference (LSD) test at *P* = 0.05 to discern differences in number of eggs and nymphs consumed under light and dark conditions. Data on larval survival to pupation and adult eclosion were analyzed by using the GLIMMIXMACRO model with a logit link function to transform data (SAS Institute, 2012). The data for egg and larval development time (days), fecundity, fertility (percent eggs hatched and the number of larvae per female), adult longevity, nymphal consumption by predators in the greenhouse and field experiments were analyzed by PROC GLM and treatment means separated using LSD.

## Results

### Feeding of *S. barberi* on Eggs and Nymphs of *D. citri*

There was no significant difference in the consumption of eggs and nymphs by *S. barberi* between light and dark condition (*P* > 0.05) except for eggs at 12 h (*F*_1_ = 4.35, *P* = 0.05) when consumption rate averaged 15.90 ± 2.4 in light and 9.70 ± 1.7 in the dark (Table 1).

**TABLE 1:** Number (Mean ± SE) of eggs and small or large nymphs of *Diaphorina citri* consumed by adult *Sympherobius barberi* during 24 h under light or dark conditions.

Diet	6 h		12 h		24 h	
	Light	Dark	Light	Dark	Light	Dark
Eggs	8.10 ± 2.27 a	7.60 ± 1.56 a	15.90 ± 2.42 a	9.70 ± 1.72 b	20.00 ± 3.20 a	19.40 ± 1.34 a
Small nymphs	19.50 ± 0.27 a	19.00 ± 0.26 a	19.90 ± 0.10 a	20.00 ± 0.00 a	20.00 ± 0.00 a	20.00 ± 0.00 a
Large nymphs	12.00 ± 2.21 a	14.10 ± 1.13 a	17.70 ± 1.20 a	16.60 ± 0.95 a	19.00 ± 0.98 a	19.00 ± 0.60 a

*Forty-eight-hour old adults received from a commercial source were reared on nectar honey and starved for 24 h prior to the test. Means within rows followed by the same letters are not significantly different between light and dark conditions at a particular time (P > 0.05).*

### Development of *S. barberi* on *D. citri* and *E. kuehniella* Diets

The development time of the eggs from parents reared on the diets of *E. kuehniella, D. citri*, and nectar honey did not differ and averaged 5 days among the three diets (*P* > 0.05, Table 2). Larvae reared on the same three diets differed significantly in their development time to pupation (*F*_2_,_27_ = 39.54, *P* < 0.001, Table 2). Development time was prolonged on the diet of *D. citri* (15.70 ± 0.58 days), followed by the diets of *E. kuehniella* (12.40 ± 0.27 days) and nectar honey (10.30 ± 0.40 days). Larval survival to pupation was 100% on the diet of *E. kuehniella* and 70% on *D. citri* and none from those on nectar honey pupated. Pupal stage lasted 11.43 ± 0.43 days for those on the diet of *D. citri* and 9.80 ± 0.61 days for those on *E. kuehniella* and did not differ statistically (*P* > 0.05, Table 2). Adult emergence from the pupae was 100% on the diets of *D. citri* and *E. kuehniella*. There was no significant difference in adult longevity when tested on the diets of eggs of *E. kuehniella* and eggs or nymphs of *D. citri* (*P* > 0.05, Table 3). The provision of honey with the diet of *D. citri* did not improve adult longevity (*P* > 0.05, Table 3).

**TABLE 2:** Development time (Mean ± SE) of *Sympherobius barberi* on diets of *Diaphorina citri, Ephestia kuehniella*, and nectar honey.

Diet	Egg stage (days)	Larval stage (days)	Pupal stage (days)
*E. kuehniella*	5.00 ± 0.09 a	12.40 ± 0.27 b	9.80 ± 0.61 a
*D. citri*	5.04 ± 0.09 a	15.70 ± 0.58 c	11.43 ± 1.43 a
Nectar honey	5.00 ± 1.00 a	10.30 ± 0.40 a	—–

*Means in columns followed by the same letters are not significantly different (P > 0.05).*

**TABLE 3:** Longevity (Mean ± SE) of adult *Sympherobius barberi* on diets of *Diaphorina citri, Ephestia kuehniella* and nectar honey alone or mixed.

Experiment 1		Experiment 2	
Diet	Longevity (days)	Diet	Longevity (days)
*D. citri* nymphs	18.40 ± 0.964 a	Nectar honey	16.00 ± 1.168 a
*D. citri* eggs	19.15 ± 1.230 a	Honey + *D. citri* (eggs + nymphs)	15.95 ± 1.243 a
*E. kuehniella* eggs	21.45 ± 1.276 a	*D. citri* (eggs + nymphs)	12.65 ± 1.240 a

*Forty-eight-hour old adults received honey on a paper strip, eggs of E. kuehniella on the surface of the petridish, and eggs or nymphs of D. citri on the shoot of M. paniculata. Means with the same letters within a column are not significantly different (P > 0.05).*

**FIGURE 3:**
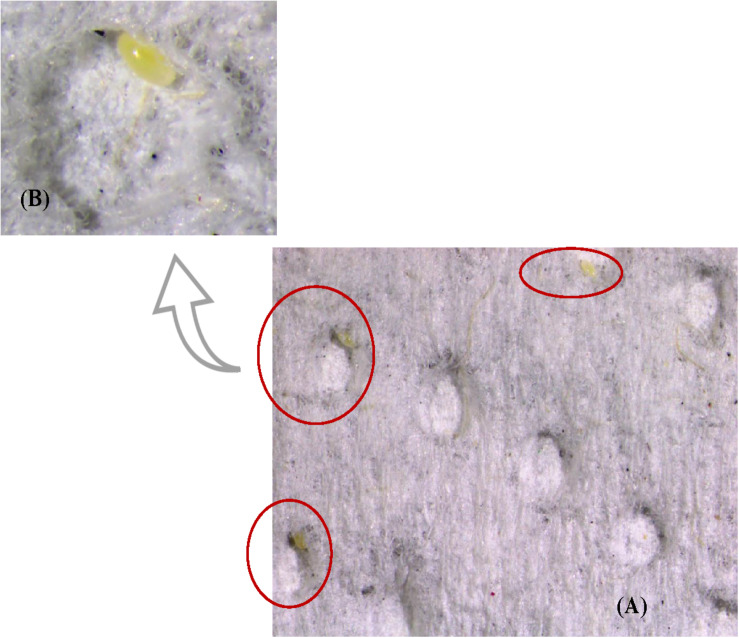
**(A)** Eggs laid by *S. barberi* on dimpled white paper. Slide photo was taken by using an Olympus SZX16 stereo microscope fixed with a DPI-21 digital camera. **(B)** Magnified view of a single dimple with egg.

In the experiment to test female oviposition preference for substrate, 64% of total eggs were laid on dimpled white paper and 36% on the remaining five substrates which included black paper, orange jasmine leaves, citrus leaves, eggplant leaves and cantaloupe leaves (Table 5 and Figures 3A,B). An average of 27 eggs per female were laid during the observation time of 1 week.

**TABLE 4:** Fecundity and fertility (Mean ± SE) of *Sympherobius barberi* on diets of *Diaphorina citri, Ephestia kuehniella* and nectar honey over 3 weeks.

Diet	Fecundity (no. of eggs/female)	Fertility (% eggs hatched)	Fertility (no. of larvae/female)
*E. kuehniella*	30.13 ± 7.18 b	51.64 ± 6.03 a	13.20 ± 3.07 a
*D. citri*	25.47 ± 2.84 b	64.96 ± 6.07 b	14.67 ± 1.19 a
Nectar Honey	0.27 ± 0.15 a	—-	—-

*Forty-eight-hour old adults were separated by distinguishing their genital characters to establish male and female pairs. Means with the same letters within a column are not significantly different (P > 0.05).*

**TABLE 5:** Percent of eggs laid on six different substrates by *S. barberi* provided with the mixed diet of *D. citri*, nectar honey and *E. kuehniella*.

No.	Substrate	Percent eggs laid
1	Dimpled white paper	64
2	Black paper	11
3	Cantaloupe leaves	9
4	Orange jasmine leaves	7
5	Eggplant leaves	5
6	Citrus leaves	4

### Reproduction of *S. barberi* on *D. citri* and *E. kuehniella* Diets

An average of 25–30 eggs laid per female did not differ significantly between the diets of *D. citri* and *E. kuehniella*; however, were significantly more than few laid on honey (*F*_2_,_42_ = 12.522, *P* < 0.001, Table 4). A significantly higher percentage of eggs hatched on the diet of *D. citri* (65%) compared to *E. kuehniella* (52%) (Table 4). There was no significant difference in the number of larvae per female (*P* > 0.05, Table 4).

### Effect of *S. barberi* Predation on *D. citri* in Potted Orange Jasmine Plants in the Greenhouse and on Citrus Trees in the Field

**FIGURE 4:**
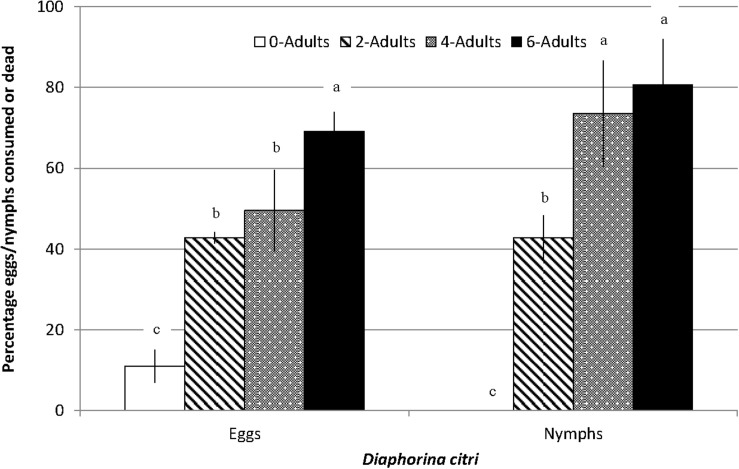
Impact of different release rates of *S. barberi* adults on eggs/nymphal colonies of *D. citri* developing on orange jasmine plants caged in the green house. Significant reduction in *D. citri*, averaging 43–81% across three release rates compared to zero-10% in control. Columns sharing the same letter within eggs or nymphs are not significantly different (*P* > 0.05).

**FIGURE 5:**
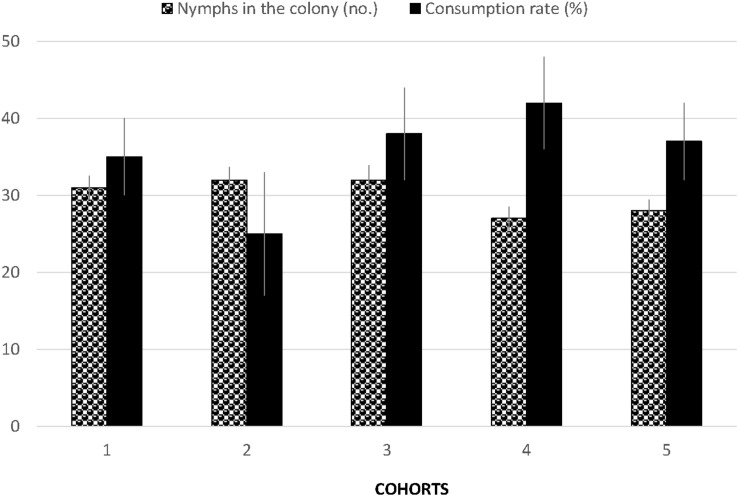
Mean number of *D. citri* nymphs in the colonies (Black and white columns) and percentage consumed (Black columns) by *S. barberi* in five cohorts. The first three cohorts contained 10 colonies each followed by 7 and 5 colonies in the 4^*th*^ and 5^*th*^ cohort, respectively. Each cohort lasted 2–3 days when the same *S. barberi* were shifted to new colonies.

The number of *D. citri* eggs on potted orange jasmine plants were significantly reduced in the cages where *S. barberi* were released compared to no release control cages (*F*_3_,_15_ = 23.31, *P* = 0.0001, Figure 4). Most reduction of 69% was observed in the treatment where six adults were released per cage, followed by 50 and 43% in the treatments with four and two adults per cage, respectively. Significant reduction in nymphs was also observed with the release of *S. barberi* on the potted orange jasmine plants in the cages compared with control (*F*_3_,_15_ = 16.45, *P* = 0.0005, Figure 4). Most reduction of 81% at high release density did not differ from 74% at medium release density, although both these reductions were significantly more than 43% observed at low release density. There was a trend of increased reduction in *D. citri* with an increase in the rate of *S. barberi* release. Under field conditions, a significant average reduction of 35% was observed in the nymphal colonies of *D. citri* caged with *S. barberi* on citrus trees compared with only 4% in control (*F*_1_,_9_ = 29.43, *P* = 0.0004). *Diaphorina citri* reduction averaged between 25 and 42% (Figure 5), over five cohorts, which included 42 nymphal colonies and each with one *S. barberi*.

## Discussion

This first detailed investigation of *S. barberi* predation against *D. citri* demonstrates the aggressive feeding behavior of this predator on psyllid eggs and nymphs. *Sympherobius barberi* were effective in consuming all the three life stages including, eggs, small nymphs, and large nymphs of *D. citri* under both light and dark conditions. There was a gradual increase in consumption rate over time, which was more evident for eggs (40 to 95% from 6 to 24 h) and large nymphs (60 to 95% from 6 to 24 h), while 95% of the small nymphs were consumed within 6 h. The feeding on eggs was initially low and improved over time, probably due to the concealed locations where they were laid, such as newly developing unfolded leaves and predator ability to reach into those locations. For large size predators such as lacewings and ladybeetles, it is difficult to attack prey in such locations, and therefore smaller predators such as predatory mites are more useful to attack eggs and neonates in such locations (Juan-Blasco et al., 2012). The difference of 35% in the consumption rate on small and large nymphs observed at 6 h was reduced to 5–10% within 12–24 h, suggesting that predator will most likely be negatively impacting significant nymphal populations encountered under field conditions. The predator preference of prey type or stage is reported for other lacewing species and pests and could impact predation rates in the field. For example, when all instars of pea aphid were presented in equal numbers to Tasmanian lacewing *Micromus tasmaniae* adults, they killed 74–90% of the first instars, 9–26% of second instars but none of the instar third and fourth (Leathwick, 1989). Islam and Chapman (2001) observed predation rate of 10 *Brevicoryne brassicae* aphids per day by the larval stage of brown lacewing *Micromus tasmaniae*, which is half of the predation rate of adult *S. barberi* on *D. citri* nymphs in the present study, suggesting better prey suitability for the predator in the latter scenario. However, the consumption rate by larvae of *S. fallax* on nymphal stages of a mealybug, *Pseudococcus longispinus*, was higher in another study (Gillani et al., 2009). We tested *S. barberi* against *D. citri* in two age groups. The successful feeding on young and mature nymphs containing 2–3 instar and 4–5 instar, respectively, followed by the experiment showing successful larval development in a diet of mixed instars indicate that they will be able to feed across all instars. Additionally, the developing colonies of *D. citri* nymphs in the field contain multiple instars which provide *S. barberi* enough choices to reduce pest population. In some situations, citrus shoots get colonized by more than one pest such as aphids and psyllids, and therefore, testing prey preference between species will be another interesting subject for a future investigation.

Larval survival of *S. barberi* to pupation on the diet of *D. citri* nymphs averaged 70% compared with 100% on the diet of frozen eggs of *E. kuehniella*. The larval development prolonged on the diet of *D. citri* nymphs compared to *E. kuehniella* eggs. The *Ephestia* eggs is a typical diet used for commercial rearing of several predators, which support maximum survival in most cases (Qureshi and Stansly, 2011; Khan et al., 2016), whereas *D. citri* was a new prey for the *S. barberi.* The presence of wing buds in the late instars nymphs of *D. citri* may have caused some deterrence for the *S. barberi* larvae and reduced the consumable body contents resulting in predator spending more time and energy in handling nymphs thus prolonged development and reduced survival in some cases. However, the pupation time of *S. barberi* did not differ between the diets of *D. citri* and *E. kuehniella*, and survival was 100% on both diets. The total development time from egg hatch to adult emergence was 26–28 days for *S. barberi* in the present study. Pacheco-Rueda et al. (2011) reported similar development time for this predator on the diet of *Dactylopius opuntiae*. Lara and Perioto (2003) reported that several species of hemerobiids complete their life cycle in 23–40 days at 23°C, and Vargas (2007) stated 23 days life cycle for *Chrysoperla carnea* at 26°. The survival rate of 70% on *D. citri* in the present study was better than some much lower rates reported for other Hemerobiids such as 2–25% for *M. tasmaniae* (Syrett and Penman, 1981), and 30% for *Hemerobius pacificus* (Neuenschwander, 1975) which most likely were impacted by rearing conditions including prey, condensation, and cannibalism. When reared individually, as in the present study, the survival of *M. tasmaniae* was in the range of 83–100% (Leathwick, 1989).

Pacheco-Rueda et al. (2011) observed an average adult longevity of 39 days for *S. barberi* on the diet of *Dactylopius opuntiae* and average of 1.89 eggs per day per female. In the present study, *S. barberi* adults evaluated in the two experiments lived between 13 and 19 days, on the diet of *D. citri*, and female produced an average of 25 eggs during a 3-week period, the latter not very different from the previous study. The difference in longevity may be due to the differences in prey and experiment conditions. The 65% of the eggs produced by *S. barberi* on the *D. citri* diet were fertile, suggesting suitability of psyllid for the successful reproductive performance of this predator.

*Sympherobius barberi* negatively impacted *D. citri* by providing significant reduction in the psyllid populations on infested orange jasmine plants under semi-natural conditions in the screenhouse and on infested citrus trees in the field. Reduction of 43–81% in the eggs and nymphs was observed when *S. barberi* were released at the rate of 2–6 adults with 100 or fewer eggs or nymphs of *D. citri* per cage. There also was a trend of increased reduction in *D. citri* with an increase in the release rate of *S. barberi*. In the field conditions, one adult caged with an average of 28–32 nymphs provided a reduction of 35%. The evidence of *S. barberi* feeding, development, and reproduction on *D. citri* and negative impact on psyllid populations under semi-natural conditions suggest that this species may play a significant role as a predator of *D. citri*. However, open releases and evaluations are needed in the groves. Legaspi et al. (1996) investigated the field release of *Chrysoperla rufilabris* in the large field cages in watermelon fields using densities of 10, 25, and 50 larvae per cage. They reported approximately 35% more whiteflies in control with no release over the entire season as compared to the predator treatment with the highest whitefly counts. Similarly, Daane et al. (1993) observed a 35% reduction of leafhopper *Erythroneura variabilis* in the vineyard fields with the release of different species of lacewings. *Sympherobius barberi* like other lacewings attacks many pests, some of which such as whiteflies, aphids etc are found in multiple crops and may help support the establishment of this predator.

## Data Availability Statement

The raw data supporting the conclusions of this article will be made available by the authors, without undue reservation.

## Author Contributions

All authors listed have made a substantial, direct and intellectual contribution to the work, and approved it for publication.

## Conflict of Interest

The authors declare that the research was conducted in the absence of any commercial or financial relationships that could be construed as a potential conflict of interest.

## References

[B1] Balduf W. V. (1939). *The Bionomics of Entomophagous Insects, Part II.* New York, NY: John S. Swift Co. Inc, 384.

[B2] Catling H. D. (1970). Distribution of the psyllid vectors of citrus greening disease, with notes on the biology and bionomics of *Diaphorina citri*. *FAO Plant Protect. Bull.* 18 8–15.

[B3] Cutright C. R. (1923). Life history of *Micromus posticus* Walker. *J. Econ. Entomol.* 16 448–456. 10.1093/jee/16.5.448

[B4] Daane K. Yokota G. Rasmussen Y. Zheng Y. Hagen K. (1993). Effectiveness of leafhopper control varies with lacewing release methods. *Calif. Agric.* 47 19–23. 10.3733/ca.v047n06p19

[B5] Gillani W. A. Copland M. Shazia R. (2009). Studies on the feeding preference of brown lacewing (Sympherobius fallax Navas) larvae for different stages of long-tailed mealy bug (Pseudococcus longispinus)(Targioni and Tozzetti). *Pakistan Entomol.* 31 1–4.

[B6] Grimaldi D. Engel M. S. (2005). *Evolution of the Insects.* Cambridge: Cambridge University Press, 755.

[B7] Halbert S. E. (1998). Entomology section. *Triology* 37 6–7.

[B8] Halbert S. E. (2005). *Pest Alert: Citrus Greening/Huanglongbing.* Gainesville, FL: Florida Department of Agriculture and Consumer Services Dept of Plant Industry.

[B9] Hussain M. A. Nath D. (1927). The citrus psylla (*Diaphorina citri*, Kuw.)[Psyllidae: Homoptera]. *Mem. Dept. Agric. India Entomol. Series* 10 1–27.

[B10] Islam S. S. Chapman R. B. (2001). “Effect of temperature on predation by Tasmanian lacewing larvae,” in *Proceedings of the New Zealand Plant Protection Conference*, Palmerston North, 244–247. 10.30843/nzpp.2001.54.3748

[B11] Juan-Blasco M. Qureshi J. A. Urbaneja A. Stansly P. A. (2012). Predatory mite *Amblyseius swirskii* (Acari: Phytoseiidae) for biological control of Asian citrus psyllid, *Diaphorina citri* (Hemiptera: Psyllidae). *Florida Entomol.* 95 543–551. 10.1653/024.095.0302

[B12] Khan A. A. Qureshi J. A. Afzal M. Stansly P. A. (2016). Two-spotted ladybeetle *Adalia bipunctata* L. (Coleoptera: Coccinellidae): a commercially available predator to control Asian Citrus Psyllid *Diaphorina citri* (Hemiptera: Liviidae). *PLoS One* 11:e0162843. 10.1371/journal.pone.0162843 27631730 PMC5025137

[B13] Killington F. J. (1937). *A Monograph of British Neuroptera, vl. 1.* London: Royal Society.

[B14] Kuwayama S. (1908). Die psylliden Japans. *Trans. Sopporo Natural History Soc. (Parts I and II)* 2 149–189.

[B15] Lara R. I. R. Perioto N. W. (2003). Hemerobiideos bioecology (Neuroptera Hemerobiidae). 70 517–523.

[B16] Leathwick D. M. (1989). *Applied Ecology of Tasmanian Lacewing Micromus tasmaniae Walker.* Ph.D. thesis, Lincoln University New Zealand, Lincoln.

[B17] Legaspi J. C. Correa J. A. Carruthers R. I. Legaspi B. C. Jr. Nordlund D. A. (1996). Effect of short-term releases of *Chrysoperla rufilabris* (Neuroptera: Chrysopidae) against silverleaf whitefly (Homoptera: Aleyrodidae) in field cages. *J. Entomol. Sci.* 31 102–111. 10.18474/0749-8004-31.1.102

[B18] Lima A. C. (1947). Insetosdo Brasil, Homopteros. Ser. Didat .4Esc. *Nac. Agron.* 3:327.

[B19] MacLeod E. G. Stange L. A. (2005). Brown Lacewings (of Florida) (Insecta: Neuroptera: Hemerobiidae). *UF University of Florida IFAS Extension* 225 1–6.

[B20] Miller G. L. Cave R. D. (1987). Bionomics of *Micromus posticus* (Walker) (Neuroptera: Hemerobiidae) with descriptions of the immature stages. *Proc. Entomol. Soc. Washington* 89 776–789.

[B21] Monserrat V. J. Marín F. (1996). Plant substrate specificity of Iberian hemerobiidae (Insecta: Neuroptera). *J. Natural History* 30 775–787. 10.1080/00222939600770401

[B22] Neuenschwander P. (1975). Influence of temperature and humidity on the immature stages of *Hemerobius pacijicus*. *Environ. Entomol.* 1 215–220. 10.1093/ee/4.2.215

[B23] New T. R. (1975). The biology of Chrysopidae and Hemerobiidae (Neuroptera), with reference to their usage as biocontrol agents: a review. *Trans. R. Entomol. Soc. Lond.* 127 115–140. 10.1111/j.1365-2311.1975.tb00561.x

[B24] New T. R. (2001). “Introduction to the Neuroptera: what are they and how do they operate,” in *Lacewings in the Crop Environment*, ed. McEwen P. K. (Cambridge: Cambridge University Press), 3–5. 10.1017/cbo9780511666117.002

[B25] Oswald J. D. (1993). Phylogeny, taxonomy, and biogeography of extant silky lacewings (Insecta: Neuroptera: Psychopsidae). *Memoirs Am. Entomol. Soc. (USA)* 40 1–65.

[B26] Oswald J. D. (1994). A new phylogenetically basal subfamily of brown lacewings from Chile (Neuroptera: Hemerobiidae). *Insect Syst. Evol.* 25 295–302. 10.1163/187631294x00090

[B27] Pacheco-Rueda I. Lomelí-Flores J. R. Rodríguez-Leyva E. Ramírez-Delgado M. (2011). Ciclo de vida y parámetros poblacionales de *Sympherobius barberi* Banks (Neuroptera: Hemerobiidae) criado con *Dactylopius opuntiae* Cockerell (Hemiptera: Dactylopiidae). *Acta Zool. Mexicana* 27 325–340. 10.21829/azm.2011.272756

[B28] Penny N. D. (1977). Lista de megaloptera, neuroptera e raphidioptera do México, América Central, ilhas Caraíbas e América do Sul. *Acta Amazonica* 7 5–61. 10.1590/1809-43921977074s005

[B29] Penny N. D. Adams P. A. Stange L. A. (1997). Species catalog of the neuroptera, megaloptera, and raphidioptera of America North of Mexico. *Proc. Calif. Acad. Sci.* 50 39–114.

[B30] Qureshi J. A. Stansly P. A. (2011). Three homopteran pests of citrus as prey for the convergent ladybeetle *Hippodamia convergens*: suitability and preference. *Environ. Entomol.* 40 1503–1510. 10.1603/en11171 22217767

[B31] SAS Institute (2012). *Release 2012.* Cary, NC: SAS Institute.

[B32] Sato T. Takada H. (2004). Biological studies on three Micromus species in Japan (Neuroptera: Hemerobiidae) to evaluate their potential as biological control agents against aphids: 1. Thermal effects on development and reproduction. *Appl. Entomol. Zool.* 39 417–425. 10.1303/aez.2004.417

[B33] Syrett P. Penman D. R. (1981). Development threshold temperatures for the brown lacewing, *Micromus tasmaniae* (Neuroptera: Hemerobiidae). *N.Z. J. Zool.* 8 281–283. 10.1080/03014223.1981.10427967

[B34] Texeira D. C. Ayres A. J. Kitajima E. W. Tanaka F. A. O. Danet J. L. Jagouiex-Eveillard S. (2005). First report of a huanglongbing-like disease of citrus in São Paulo state, Brazil, and association of a new Liberibacter species,“Candidatus Liberibacter americanus,” with the disease. *Plant Dis.* 89 107. 10.1094/pd-89-0107a 30795297

[B35] Vargas E. U. (2007). Development, Survival and Fecundity of Chrysoperla carnea Stephens and Chrysoperla comanche Banks Reared Eggs of Ephestia kuehniella (Olivier). Master’s thesis, Chapingo University, Mexico.

[B36] Yayla M. Satar S. (2012). Temperature influence on development of *Sympherobius pygmaeus* (Rambur) (Neuroptera: Hemerobiidae) reared on *Planococcus citri* (Risso) (Hemiptera: Pseudococcidae). *Türkiye Entomol. Dergisi* 36 11–22.

